# Apolipoprotein A-IV Cardiac Amyloidosis as a Diagnostic Pitfall: Beyond the Noninvasive Algorithm

**DOI:** 10.1016/j.jaccas.2026.108669

**Published:** 2026-05-10

**Authors:** Anne J. Koppelaar, Peter-Paul Zwetsloot, Jan H. von der Thüsen, King H. Lam, Janet A. Gilbertson, Julian D. Gillmore, Michelle Michels, Alexander Hirsch

**Affiliations:** a Department of Cardiology, Amphia Hospital, Breda, the Netherlands; b Department of Cardiology, Cardiovascular Institute, Thoraxcenter, Erasmus Medical Center, Rotterdam, the Netherlands; c Department of Pathology, Erasmus Medical Center, Rotterdam, the Netherlands; d National Amyloidosis Centre, University College London, Royal Free Hospital, London, United Kingdom; e Department of Radiology and Nuclear Medicine, Erasmus Medical Center, Rotterdam, the Netherlands

**Keywords:** cardiac magnetic resonance, cardiomyopathy, imaging

## Abstract

**Background:**

Cardiac amyloidosis is frequently underdiagnosed, and rarer forms (eg, nontransthyretin amyloidosis, light chain amyloidosis) can be challenging to uncover.

**Case Summary:**

A 71-year-old man with reduced kidney function presented with new-onset heart failure, concentric left ventricular hypertrophy, and an “apical sparing” strain pattern. Cardiac magnetic resonance confirmed an infiltrative phenotype with suggestive late gadolinium enhancement and parametric mapping pattern. However, bone scintigraphy and monoclonal protein screening were negative; therefore, an endomyocardial biopsy was performed. After negative immunohistochemistry, mass spectrometry-based proteomics finally identified apolipoprotein A–IV amyloidosis.

**Discussion:**

Apolipoprotein A–IV cardiac amyloidosis represents a critical diagnostic blind spot and requires molecular proteomics for definitive typing.

**Take-Home Messages:**

A pathognomonic cardiac magnetic resonance phenotype for cardiac amyloidosis, despite negative bone scintigraphy and monoclonal protein screening, should prompt invasive investigation to identify rare amyloid types. Endomyocardial biopsy with mass spectrometry remains the definitive diagnostic technique for amyloid typing in such cases.

## Visual Summary

**Figure:**
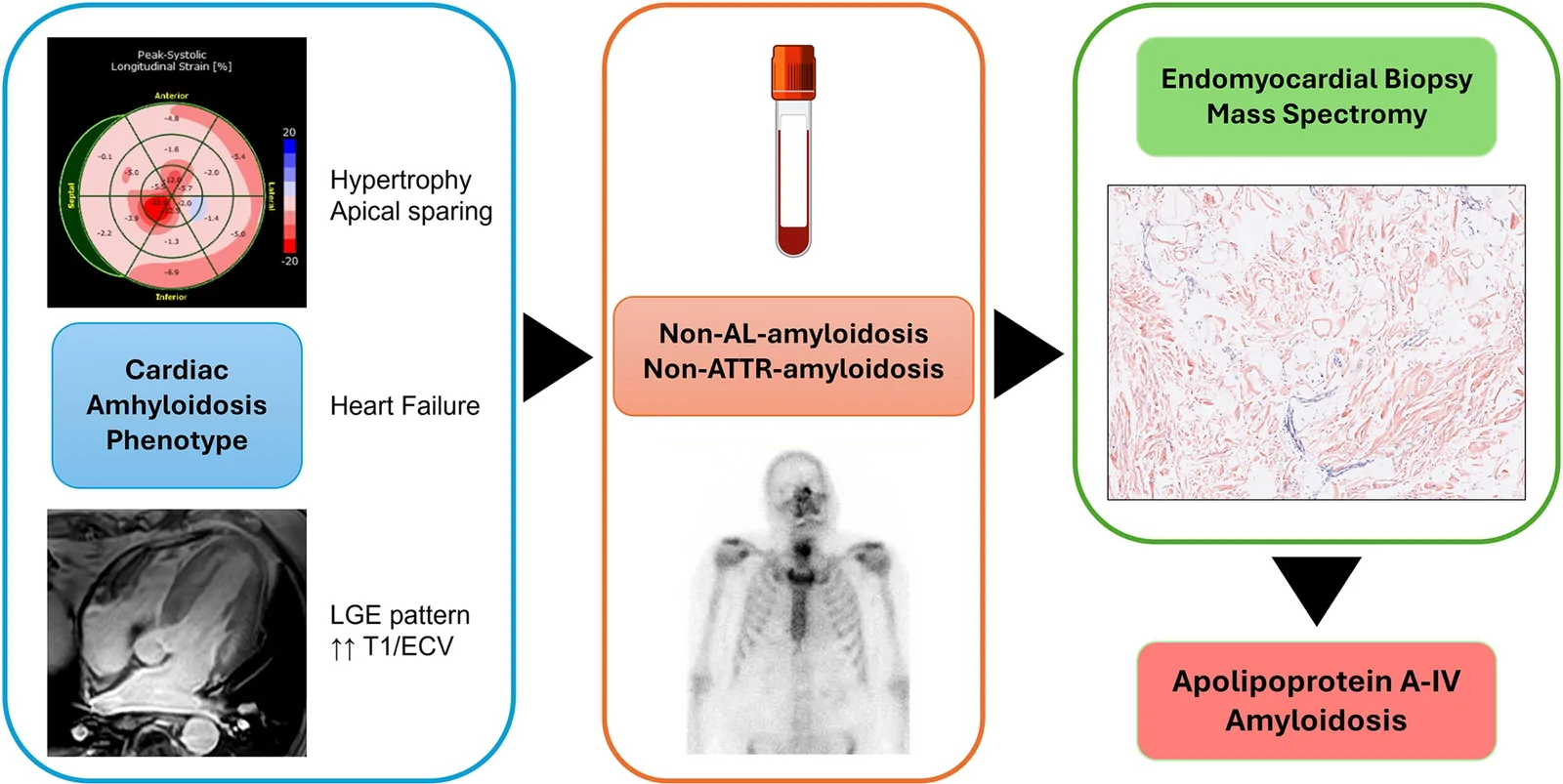

## History of Presentation

**Figure 1:**
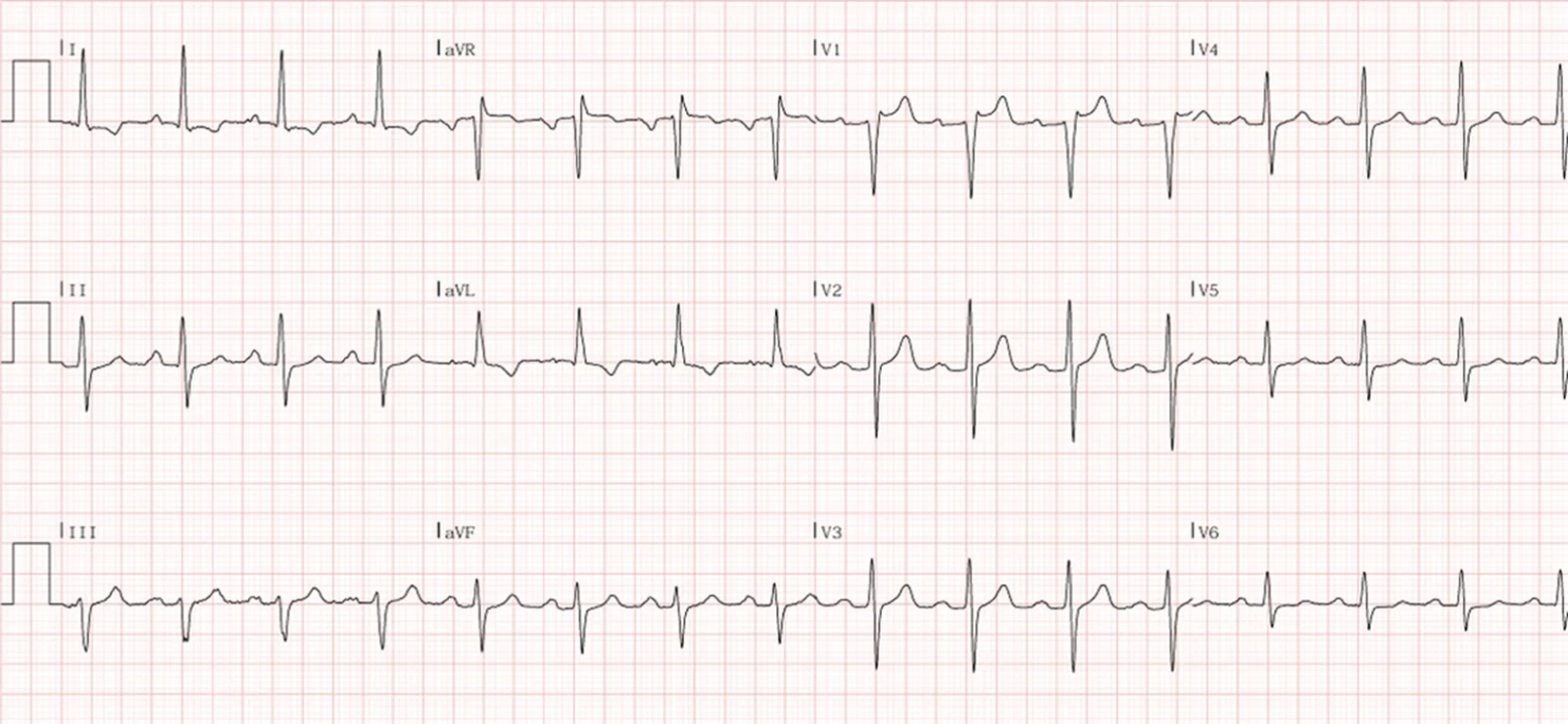
Electrocardiogram The electrocardiogram showed sinus rhythm with a slightly prolonged PR interval (210 milliseconds) and low voltage relative to the severity of left ventricular hypertrophy on echocardiography.

**Figure 2:**
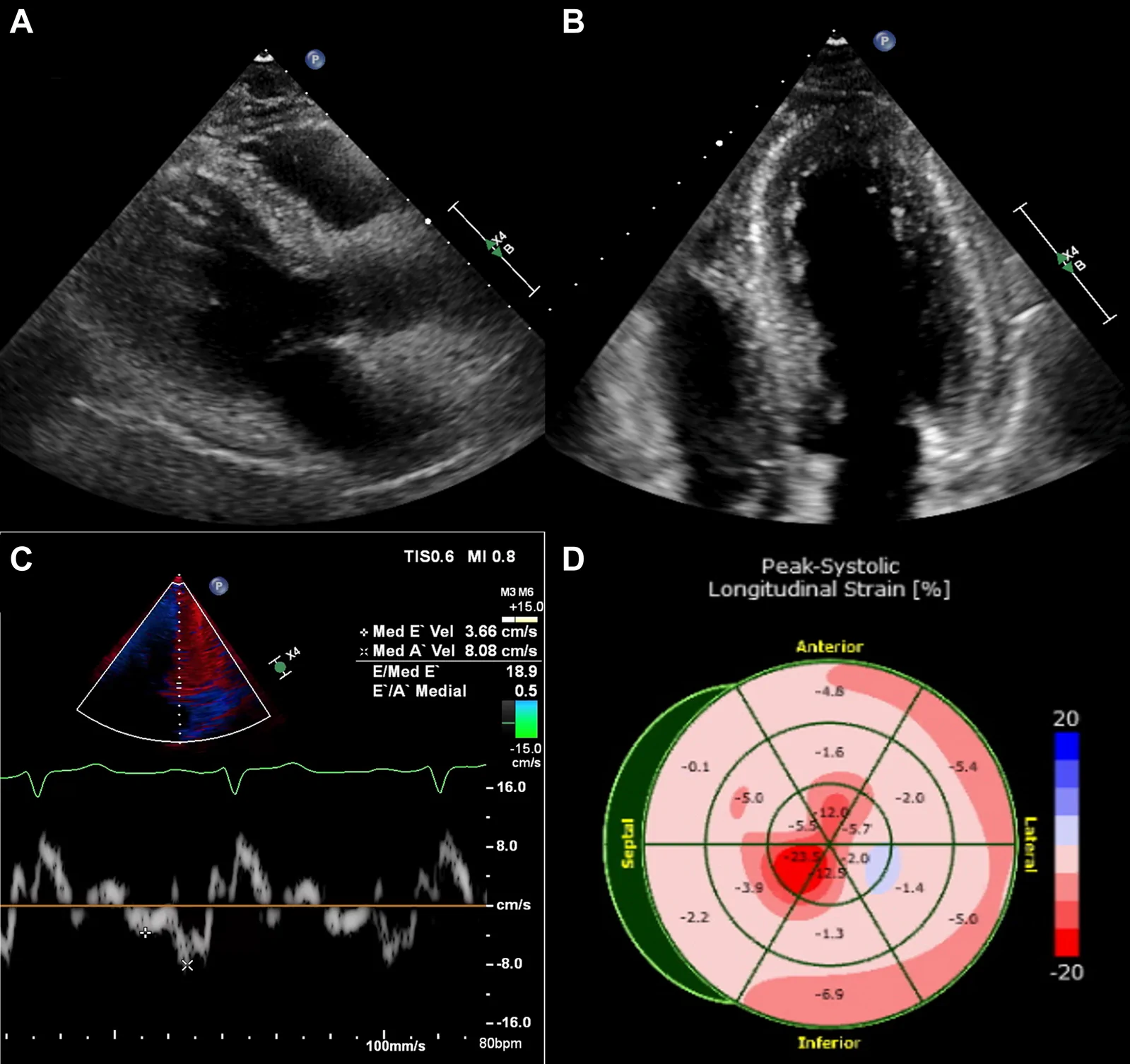
Echocardiography Echocardiography showed concentric left ventricular hypertrophy without relevant valvular disease (parasternal long-axis view) (A). Interventricular septum of 17 mm and inferolateral wall of 12 mm, resulting in an increased relative wall thickness of 0.63 and an indexed left ventricular mass of 123 g/m^2^ (apical 4-chamber view) (B). Diastolic dysfunction was present with an E/A ratio of 0.8, medial e' of 3.7 cm/s, and an elevated E/e' ratio of 18.9 (C). Strain imaging showed reduced systolic longitudinal strain (−5.2%) with apical sparing (D).

A 71-year-old man was referred to our tertiary medical center after he presented at a general hospital with congestive heart failure and elevated cardiac troponin. The initial electrocardiography showed sinus rhythm with a slightly prolonged PR interval (210 milliseconds) and a lack of left ventricular hypertrophy (Figure 1). Significantly elevated cardiac troponins initially raised concern for myocardial ischemia, but coronary angiography revealed no obstructive coronary artery disease. Echocardiography showed concentric left ventricular wall thickening with preserved ejection fraction (65%). Diastolic dysfunction was present, and global longitudinal strain was severely reduced with apical sparing (Figure 2). Laboratory analyses showed elevated N-terminal pro–B-type natriuretic peptide of 358 pmol/L (upper limit of normal: 15). The patient exhibited NYHA functional class III symptoms and was managed with a regimen of angiotensin-converting enzyme inhibitors, beta-blockers, and oral glucose-lowering agents.

## Past Medical History

His history was notable for bilateral carpal tunnel syndrome and extensive orthopedic morbidity, including bilateral total hip replacements, followed by a right total knee replacement. He has had a long-standing history of renal dysfunction with significant proteinuria for >10 years. His comorbidities included hypertension and type 2 diabetes mellitus. His family history was negative for cardiomyopathies, sudden cardiac death, or amyloidosis.

## Differential Diagnosis

The restrictive cardiomyopathy phenotype, characterized by preserved ejection fraction, severe concentric left ventricular thickening, and an apical sparing pattern, strongly suggests cardiac amyloidosis (CA). Given the extensive extracardiac red flags, transthyretin amyloidosis (ATTR) CA was considered the primary clinical suspicion.

However, the definitive typing of the amyloid deposits remained critical because the therapeutic implications differed drastically. Light chain (AL) amyloidosis had to be rigorously excluded, especially given the patient's history of unexplained proteinuria and renal dysfunction. Although the orthopedic history favored a more indolent course of ATTR amyloidosis, the significant proteinuria prompted evaluation for plasma cell dyscrasia.

Beyond CAs, other infiltrative and hypertrophic pathologies were considered, but deemed less likely. Hypertrophic cardiomyopathy, specifically the concentric phenotype, remained a potential explanation, because it can present with similar degrees of wall thickening and severe diastolic impairment. Hypertensive heart disease was also considered, but deemed less likely, because the severity of the wall thickening appeared disproportionate to the patient's hypertensive history and blood pressure measurements over time were not markedly elevated (150/72 mm Hg at presentation and 139/75 mm Hg during follow-up). Furthermore, Fabry disease was considered but deemed unlikely given the late onset and absence of characteristic extracardiac features.

## Investigations

**Figure 3:**
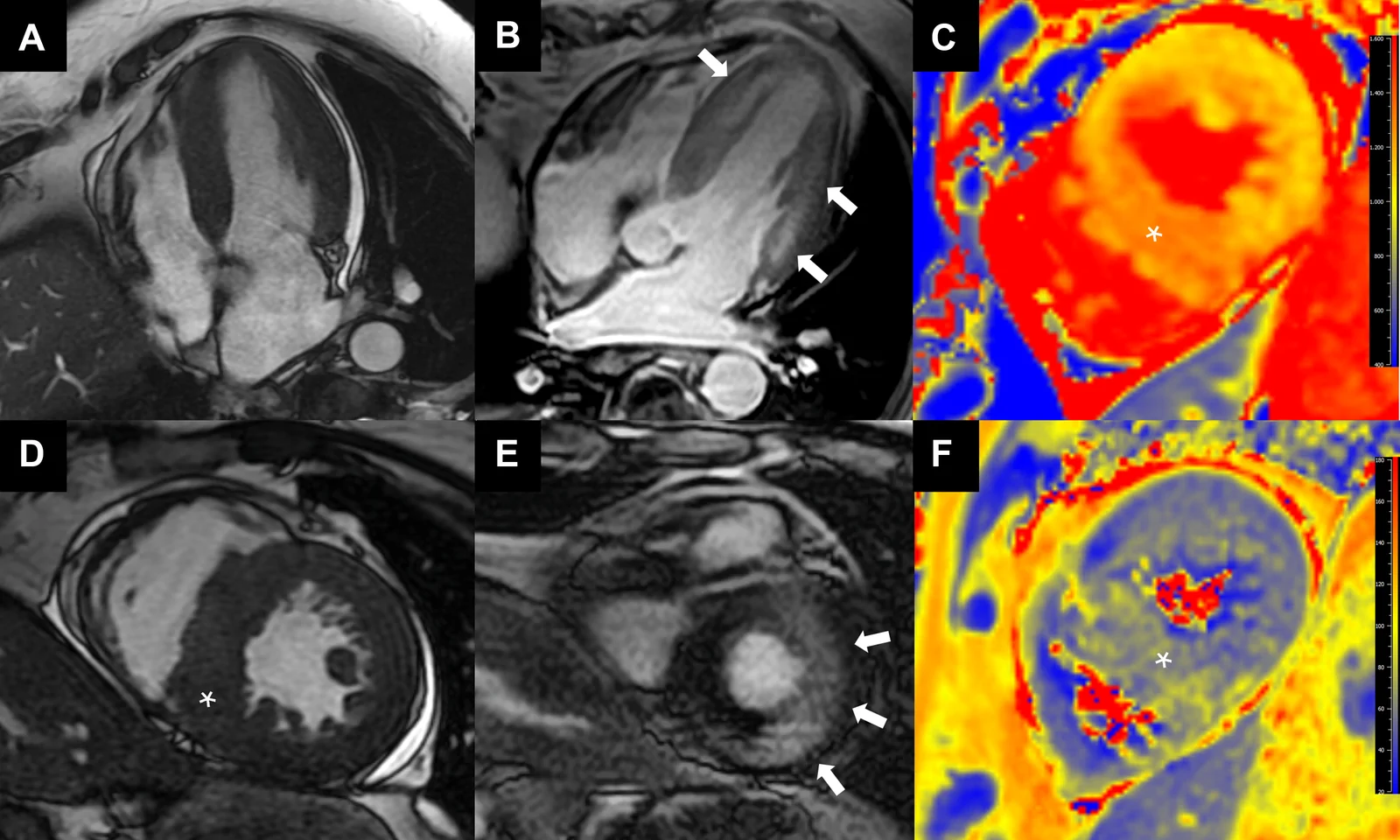
Cardiac Magnetic Resonance Cine imaging (4-chamber view [A] and short-axis view [D]) showed concentric left ventricular hypertrophy with a maximum wall thickness of 24 mm (asterisk in panel D) and mild pericardial effusion. Late gadolinium enhancement images in the 4-chamber and basal short-axis views are shown in panels B and E, respectively, demonstrating diffuse enhancement throughout the left ventricle (open arrows), with partial transmural involvement. Native T1 mapping (mid-ventricular short axis) showed clearly elevated values, especially in the septum (septal native T1: 1,277 milliseconds; normal value: <1,020 milliseconds) (asterisk in panel C). There were also elevated T2 values (septal T2 value: 67 milliseconds; normal value: <56 milliseconds) (asterisk in panel F).

**Figure 4:**
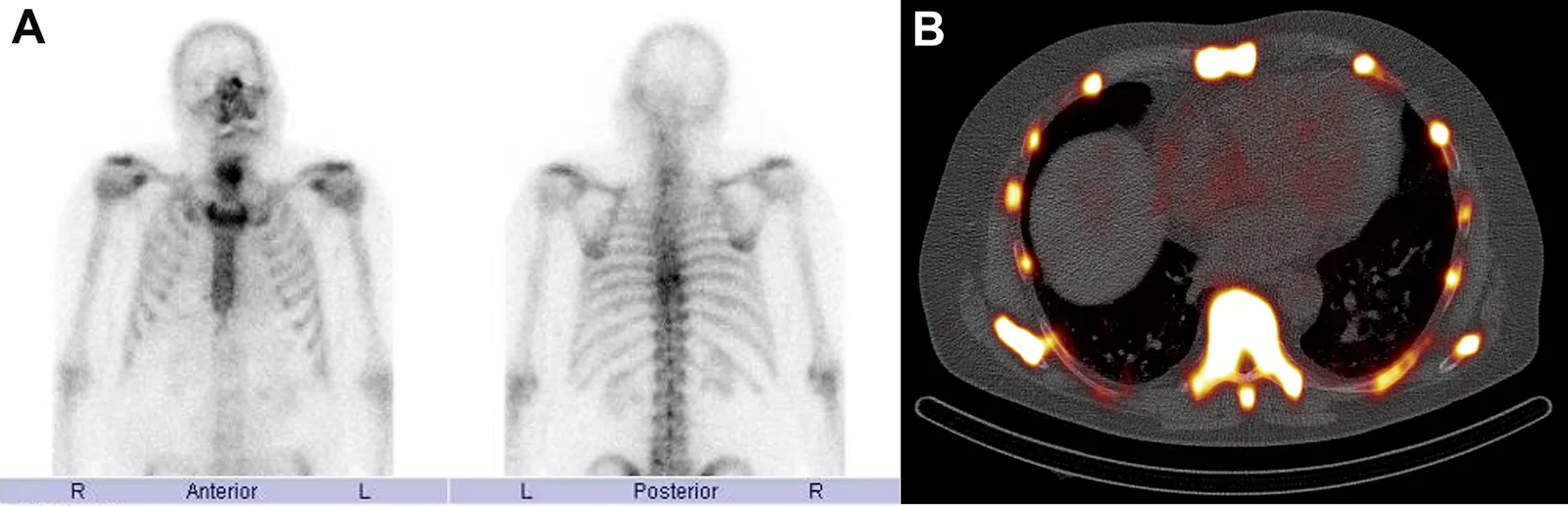
Bone Scintigraphy Planar imaging (A) and single-photon emission computed tomography (B) did not show any cardiac uptake (Perugini grade 0) on technetium-labeled bone scintigraphy.

Cardiac magnetic resonance (CMR) was strongly suggestive of CA with a maximal wall thickness of 24 mm, interatrial septal hypertrophy, mild pericardial effusion, and diffuse, partially transmural, late gadolinium enhancement (LGE) with increased native T1 and extracellular volume (mid-ventricular septum: 40%) (Figure 3). Laboratory screening for monoclonal gammopathy was negative, effectively ruling out AL amyloidosis. Although free AL levels were elevated (lambda: 23.0 mg/L, kappa: 46.5 mg/L), the ratio of 2.02 was consistent with severe renal impairment (estimated glomerular filtration rate [eGFR]: 22 mL/min/1.73 m^2^). Bone scintigraphy showed a Perugini grade 0, challenging the suspicion of ATTR CA (Figure 4).

**Figure 5:**
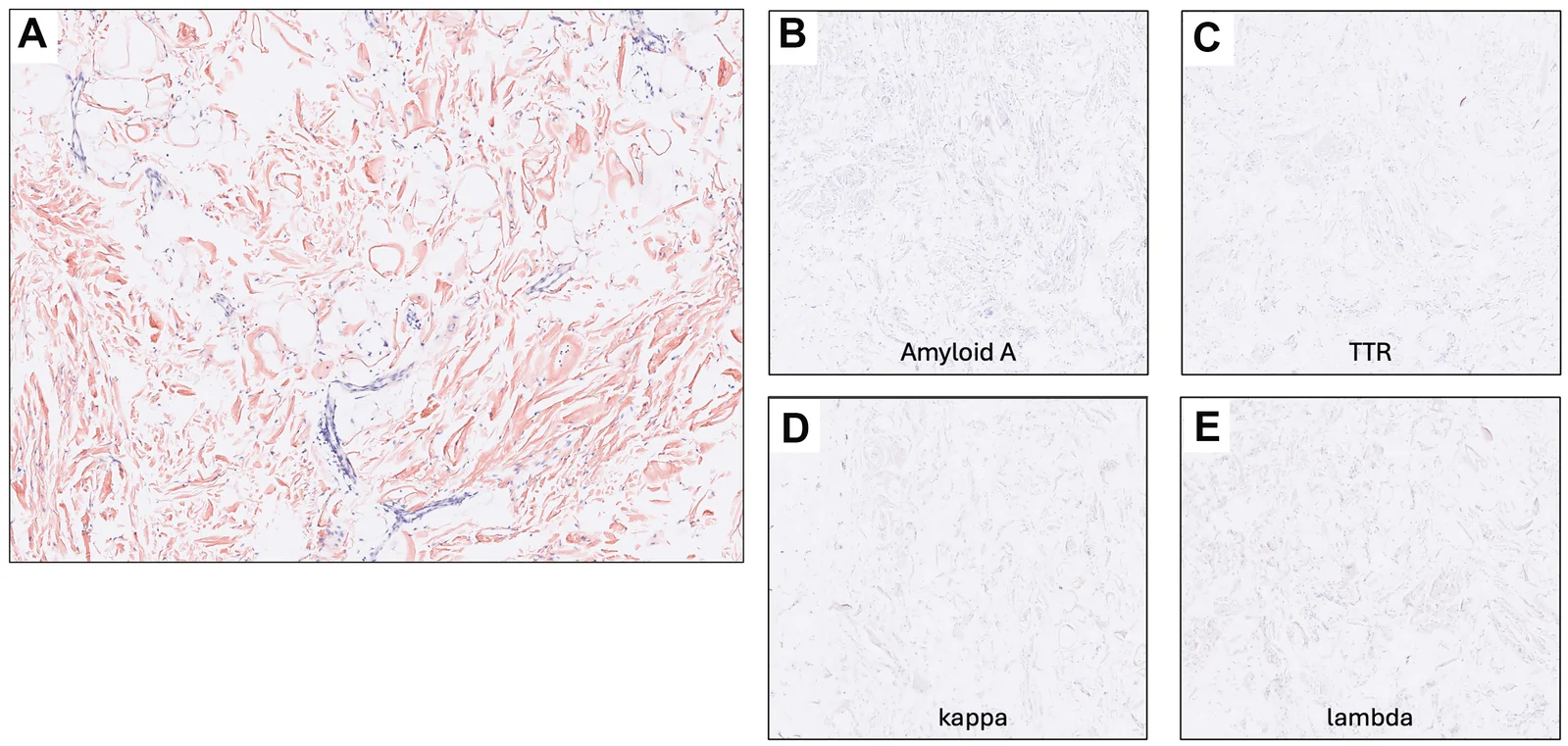
Fat Pad Biopsy and Immunohistochemistry (A) Fat pad biopsy and subsequent histochemistry revealed evident Congo red staining. Immunohistochemistry was negative for amyloid A (B), transthyretin (TTR) (C), kappa light chains (D), and lambda light chains (E).

**Figure 6:**
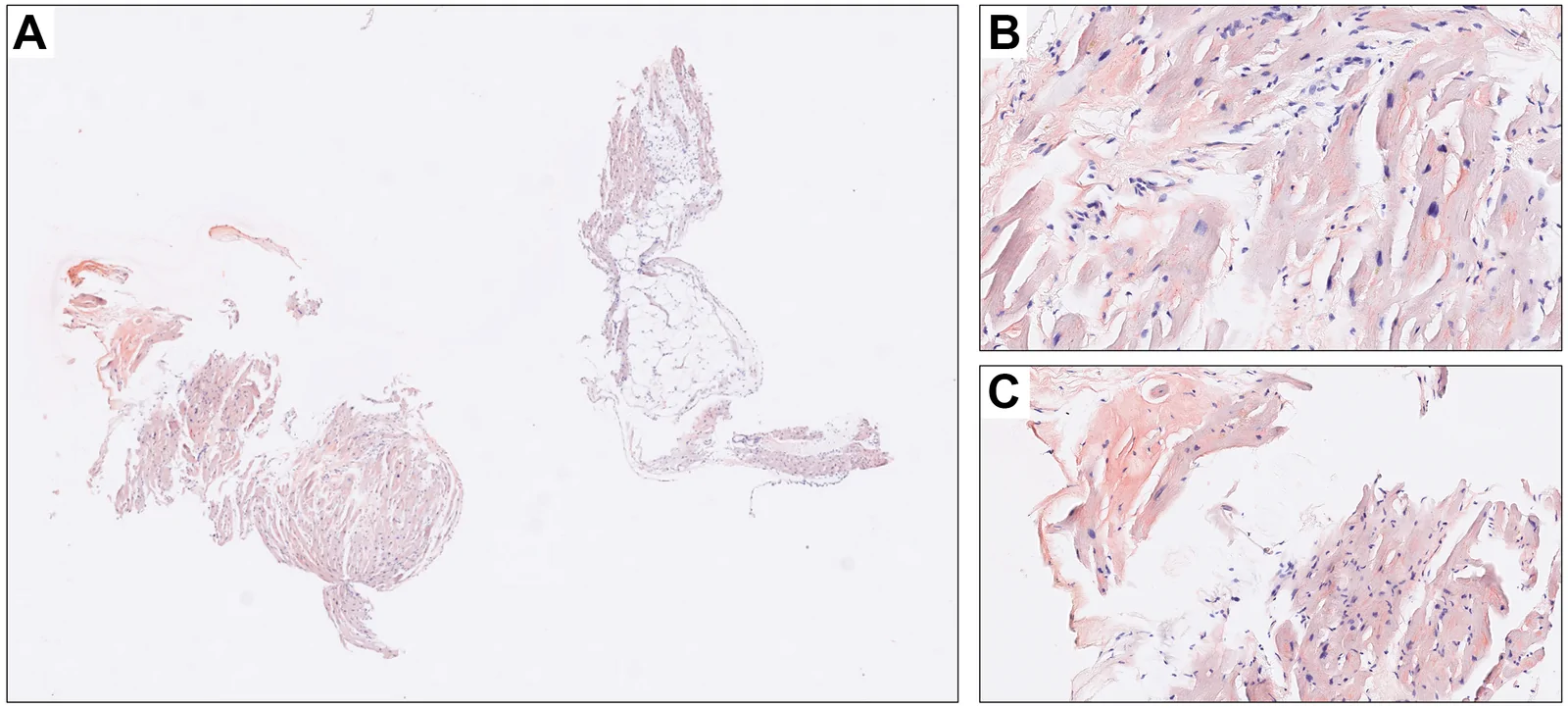
Cardiac Biopsy and Congo Red Histochemistry (A-C) Cardiac biopsy confirmed amyloid presence by Congo red staining.

Because of the profound discrepancy between CMR findings and noninvasive screening with bone scintigraphy and laboratory analyses, advanced diagnostics were pursued. Comprehensive DNA testing for hypertrophic cardiomyopathy and hereditary amyloidosis was performed, particularly because certain *TTR* variants may yield false-negative bone scintigraphy. However, DNA analysis was negative and revealed no pathogenic variants in the *TTR* or ApoA-I genes. A bone marrow biopsy did not identify a clonal disease. Concurrently, an abdominal fat pad biopsy confirmed amyloid deposition by Congo red staining (Figure 5A); however, immunohistochemistry was negative with antibodies against transthyretin (TTR), kappa and lambda ALs, and amyloid A protein (Figures 5B to 5E). Definitive characterization was subsequently sought through an endomyocardial biopsy, which confirmed cardiac amyloid deposits on Congo red staining (Figure 6). Because immunohistochemistry and immunoelectron microscopy were again negative for common amyloid subtypes, a rare variant, such as ApoA-IV amyloidosis, was suspected, necessitating mass spectrometry for proteomic confirmation. The definitive diagnosis of apolipoprotein A (ApoA)-IV amyloidosis was established through mass spectrometry–based proteomics of the myocardial tissue (performed at the National Amyloidosis Centre, London, United Kingdom).

## Management

In accordance with current guidelines for rare non-ATTR/non-AL variants, management focused on stringent non–amyloid-specific treatment.^1^ Unlike in AL amyloidosis, there is no indication for chemotherapy or stem cell transplantation. Furthermore, because the pathophysiology does not involve transthyretin, TTR stabilizers such as tafamidis and liver transplantation are not indicated. Current management consists of optimized heart failure treatment, renal preservation, and multidisciplinary follow-up. Fluid balance was maintained through individualized diuretic therapy. With an eGFR <20 mL/min/1.73 m^2^, a sodium-glucose cotransporter 2 inhibitor was not initiated.

## Outcome and Follow-Up

Although the lack of targeted therapies for ApoA-IV presents a therapeutic challenge, the definitive diagnosis nevertheless provides clarity regarding the prognosis and prevents exposure to inappropriate and potentially toxic treatments. This case illustrates that in the era of precision medicine, mass spectrometry on an endomyocardial biopsy is the final arbiter in the diagnostic work-up of this restrictive cardiomyopathy. For the patient, diagnostic clarification was particularly valuable given the potential familial implications. Given the potential heritable component reported in the current literature, our genetic department will investigate specific ApoA-IV polymorphisms.^2^ Meanwhile, the patient is relatively stable, without any progression of symptoms, heart failure exacerbations, or hospitalizations after the initial diagnosis (now 6 months later). His most recent echocardiography remained unchanged. However, his eGFR decreased to 12 mL/min/1.73 m^2^, and his N-terminal pro–B-type natriuretic peptide increased to 1,179 pmol/L. He is discussing the possibilities of dialysis with his nephrologist; however, his CA and heart failure obviously complicate this scenario. Furthermore, the patient likes his independence, and so is also very much considering a palliative track, foregoing the option of dialysis.

## Discussion

ApoA-IV is a protein mainly synthesized in the small intestine, responsible for the transport, absorption, and metabolism of lipids.^3^ Past cohort studies have deemed the prevalence of ApoA-IV CA to be <1% of all cardiac cases at the National Amyloidosis Centre in the United Kingdom (cohort between 2000 and 2021) and Mayo Clinic (cohort 2006-2015).^4^^,^^5^ With increasing recognition of AL and ATTR amyloidosis, the relative contribution of ApoA-IV CA has likely declined further. Of note, our patient already had long-lasting, slowly progressive chronic kidney disease, which is also in line with the ApoA-IV amyloidosis diagnosis. Similar to our case, ApoA-IV CA usually presents with heart failure and classical pathognomonic signs of CA on echocardiography and CMR.^4^

CMR plays a pivotal role in the diagnostic work-up of patients presenting with new-onset heart failure, particularly in the setting of unexplained left ventricular hypertrophy. In our patient, CMR was instrumental in shifting the diagnostic focus from a broad differential to CA characterized by diffuse myocardial infiltration and abnormal gadolinium kinetics (eg, difficult nulling of the myocardium). Furthermore, quantification of native T1 values and extracellular volume has been validated as a robust surrogate for histologic amyloid burden, offering superior diagnostic accuracy compared with LGE alone.^6^

CMR excluded our differential diagnosis of hypertrophic cardiomyopathy because of the LGE pattern and the absence of sarcomeric-related morphologic criteria. Furthermore, the severity of the expanded extracellular volume and the characteristic global LGE pattern were disproportionate to what would be expected in hypertensive heart disease. The high native T1 values steered the diagnosis away from Fabry disease, which is often a hallmark of sphingolipid deposition.

Importantly, current guidelines recommend performing cardiac biopsy when there is high suspicion of CA, despite negative bone scintigraphy. This is critical because certain (hereditary) ATTR-CA, such as the rare variants *Phe64Leu* and the “early onset” *Val30Met* (type B fibrils), and non-TTR types like AopA-I, ApoA-IV, and beta2-microglobin amyloidosis, frequently yield false-negative scintigraphy.^7^, ^8^, ^9^, ^10^ However, the results of immunohistochemistry are dependent on antibody availability, the natural background presence of the protein of interest (ApoA-IV is a natural chaperone protein in many amyloid deposits), and local expertise. Although Congo red staining was present in the fat pad biopsy, the immunohistochemistry was inconclusive. Therefore, we decided to proceed with an endomyocardial biopsy to obtain a higher-yield tissue sample for mass spectrometry, ensuring a definitive diagnosis in this “double-negative” scenario. Mass spectrometry–based proteomics provides an alternative method for identifying the amyloid fibril protein, including ApoA-IV, as in this case.

## Conclusions

This case identifies a critical diagnostic blind spot in managing restrictive cardiomyopathies caused by CA. A pathognomonic CMR phenotype for CA should serve as a gatekeeper in cardiomyopathy evaluation when monoclonal protein screening and bone scintigraphy are negative. Moreover, subsequent endomyocardial biopsy and mass spectrometry–based molecular proteomics enabled accurate identification of rare CA variants, including ApoA-IV, as in this case, which frequently yield false-negative bone scintigraphy results. Accurate identification is crucial to avoid ineffective or toxic AL- or ATTR-specific therapies. In the era of precision medicine, molecular proteomics has emerged as a definitive diagnostic tool, providing the clarity needed to guide management and facilitate future precision therapies in CA.

**Visual Summary:**
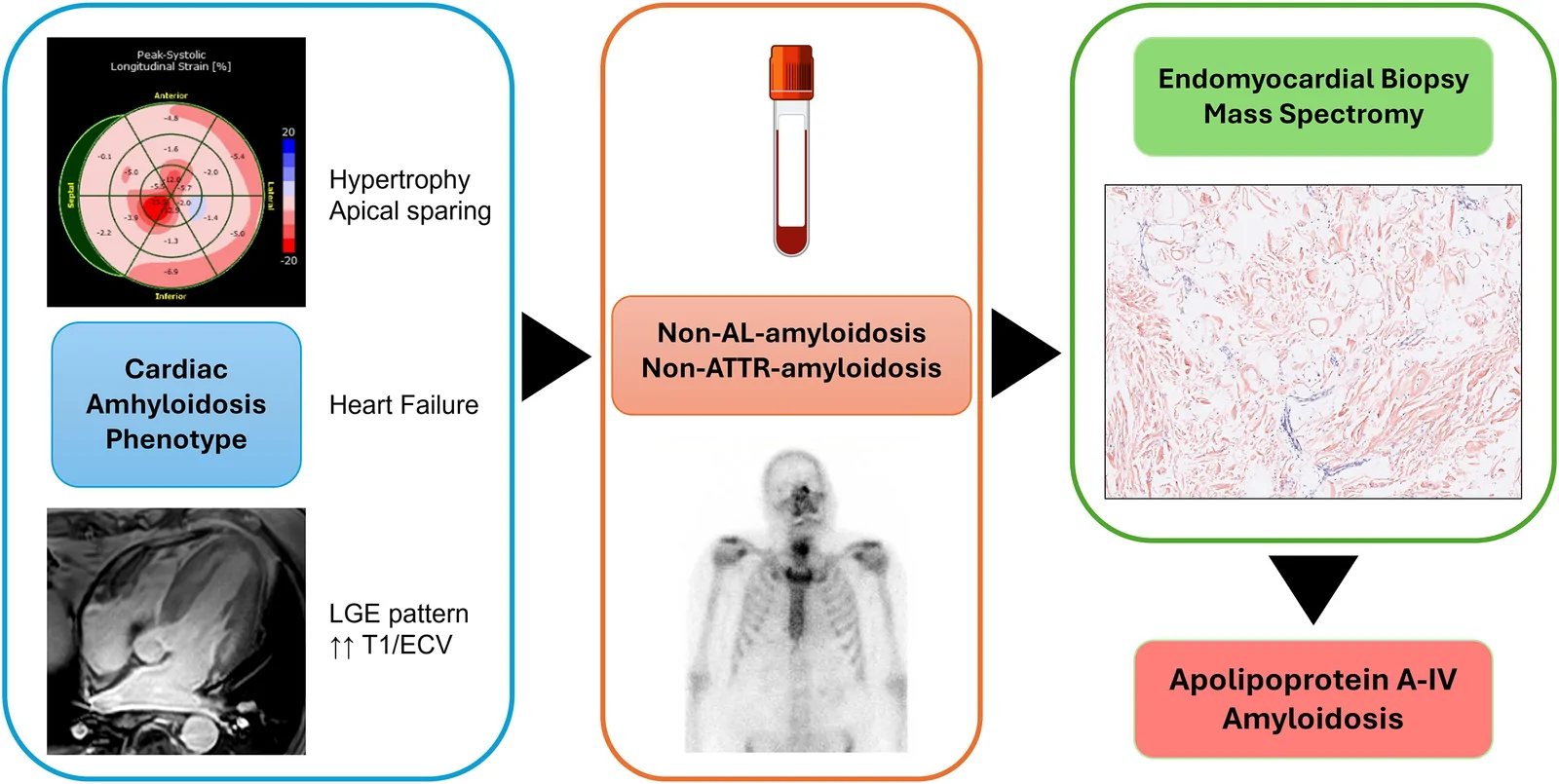
Diagnostic Blind Spot A pathognomonic imaging phenotype for cardiac amyloidosis with negative noninvasive screening, leading to the definitive diagnosis of apolipoprotein A–IV amyloidosis via mass spectrometry. AL = light chain; ECV = extracellular volume; ATTR = transthyretin amyloidosis; LGE = late gadolinium enhancement.

## Funding Support and Author Disclosures

Dr Zwetsloot has received speaker fees from Mednet, Pfizer, Bayer, and Bristol Myers Squibb; consultancy fees from Bayer, Pfizer, PHARMO, Alnylam, Novo Nordisk, Cytokinetics, and Bristol Myers Squibb; and research grants from Pfizer, AstraZeneca, and Bayer. Dr Gillmore had received consultancy for Alnylam, AstraZeneca, Alexion, Attralus, BridgeBio, Bayer, Ionis, Intellia, and Pfizer; and is a recipient of institutional grants from Alnylam and AstraZeneca. Dr Michels had received research grants from Bristol Meyers Squibb, AstraZeneca, and Bayer; and consultancy or speakers fees from Bristol Meyers Squibb, Cytokinetics, Bayer, Alnylam, Braveheart, Novo Nordisk, and Sanofi-Genzyme. Dr Hirsch has received a research grant and consultancy fees from GE Healthcare; has received speaker fees from GE Healthcare, Bayer, Bristol Myers Squibb, Siemens Healthineers, and Heartflow; and is a member of the medical advisory board of Medis Medical Imaging Systems. All other authors have reported that they have no relationships relevant to the contents of this paper to disclose.

**Take-Home Messages**

• When cardiac magnetic resonance identifies a high-confidence amyloid phenotype, a negative bone scintigraphy and monoclonal protein screening should prompt invasive investigation to identify rare amyloid types.
• Endomyocardial biopsy with mass spectrometry remains the definitive diagnostic technique for amyloid typing in such cases.
• Relying solely on noninvasive tools may lead to the underdiagnosis of rare but clinically significant amyloid variants like apolipoprotein A–IV.
